# Computed tomography hypoperfusion-hypodensity mismatch vs. automated perfusion mismatch to identify stroke patients eligible for thrombolysis

**DOI:** 10.3389/fneur.2023.1320620

**Published:** 2023-12-29

**Authors:** Peter B. Sporns, André Kemmling, Lennart Meyer, Christos Krogias, Volker Puetz, Kolja M. Thierfelder, Marco Duering, Carsten Lukas, Daniel Kaiser, Sönke Langner, Alex Brehm, Lukas T. Rotkopf, Wolfgang G. Kunz, Carolin Beuker, Walter Heindel, Jens Fiehler, Peter Schramm, Heinz Wiendl, Heike Minnerup, Marios Nikos Psychogios, Jens Minnerup

**Affiliations:** ^1^Department of Neuroradiology, Clinic of Radiology and Nuclear Medicine, University Hospital Basel, Basel, Switzerland; ^2^Department of Diagnostic and Interventional Neuroradiology, University Medical Center Hamburg-Eppendorf, Hamburg, Germany; ^3^Department of Radiology, Westfaelische Wilhelms-University of Münster and University Hospital of Münster, Münster, Germany; ^4^Department of Neuroradiology, Westpfalz-Klinikum, Kaiserslautern, Germany; ^5^Department of Neuroradiology, University Medical Center Schleswig-Holstein, Lübeck, Germany; ^6^Department of Neurology with Institute of Translational Neurology, University of Münster, Münster, Germany; ^7^Department of Neurology, St. Josef-Hospital, Ruhr University Bochum, Bochum, Germany; ^8^Department of Neurology, University Hospital Carl Gustav Carus, Dresden, Germany; ^9^Department of Radiology and Institute of Diagnostic and Interventional Radiology, University Medical Center Rostock, Rostock, Germany; ^10^Medical Image Analysis Center (MIAC) and Department of Biomedical Engineering, University of Basel, Basel, Switzerland; ^11^Institute for Stroke and Dementia Research, University Hospital, LMU Munich, Munich, Germany; ^12^Department of Neuroradiology, St. Josef-Hospital, Ruhr University Bochum, Bochum, Germany; ^13^Department of Neuroradiology, University Hospital Carl Gustav Carus, Dresden, Germany; ^14^Department of Radiology, German Cancer Research Center, Heidelberg, Germany; ^15^Department of Radiology, University Hospital, LMU Munich, Germany; ^16^Institute of Epidemiology and Social Medicine, University of Münster, Münster, Germany

**Keywords:** stroke, computed tomography, time window, thrombolysis, unknown onset stroke

## Abstract

**Background and purpose:**

Automated perfusion imaging can detect stroke patients with unknown time of symptom onset who are eligible for thrombolysis. However, the availability of this technique is limited. We, therefore, established the novel concept of computed tomography (CT) hypoperfusion-hypodensity mismatch, i.e., an ischemic core lesion visible on cerebral perfusion CT without visible hypodensity in the corresponding native cerebral CT. We compared both methods regarding their accuracy in identifying patients suitable for thrombolysis.

**Methods:**

In a retrospective analysis of the MissPerfeCT observational cohort study, patients were classified as suitable or not for thrombolysis based on established time window and imaging criteria. We calculated predictive values for hypoperfusion-hypodensity mismatch and automated perfusion imaging to compare accuracy in the identification of patients suitable for thrombolysis.

**Results:**

Of 247 patients, 219 (88.7%) were eligible for thrombolysis and 28 (11.3%) were not eligible for thrombolysis. Of 197 patients who were within 4.5 h of symptom onset, 190 (96.4%) were identified by hypoperfusion-hypodensity mismatch and 88 (44.7%) by automated perfusion mismatch (*p* < 0.001). Of 22 patients who were beyond 4.5 h of symptom onset but were eligible for thrombolysis, 5 patients (22.7%) were identified by hypoperfusion-hypodensity mismatch. Predictive values for the hypoperfusion-hypodensity mismatch vs. automated perfusion mismatch were as follows: sensitivity, 89.0% vs. 50.2%; specificity, 71.4% vs. 100.0%; positive predictive value, 96.1% vs. 100.0%; and negative predictive value, 45.5% vs. 20.4%.

**Conclusion:**

The novel method of hypoperfusion-hypodensity mismatch can identify patients suitable for thrombolysis with higher sensitivity and lower specificity than established techniques. Using this simple method might therefore increase the proportion of patients treated with thrombolysis without the use of special automated software.

The MissPerfeCT study is a retrospective observational multicenter cohort study and is registered with clinicaltrials.gov (NCT04277728).

## 1 Introduction

Patients with acute ischemic stroke can receive intravenous thrombolysis up to 4.5 h after symptom onset (1). However, up to 25% of all stroke patients have an unknown time of symptom onset because stroke occurs while sleeping or the time of onset cannot be communicated due to aphasia or a disturbed level of consciousness (2). Patients with unknown time of symptom onset who are suitable for thrombolysis can be identified by penumbral imaging, i.e., the identification of hypoperfused but potentially salvageable brain tissue (3). Such evaluation of perfusion imaging requires the application of dedicated software with limited availability (4). An additional method for the identification of patients with unknown time of symptom onset suitable for thrombolysis is a so-called “tissue clock” approach, whereby a visible lesion mismatch between diffusion-weighted imaging (DWI) and fluid-attenuated inversion recovery (FLAIR) sequences indicates a stroke within the time window of thrombolysis (5, 6). This approach is limited by the restricted availability of MRI for acute stroke triage.

Recently, in the multicenter MissPerfeCT study, we have established the new and simple CT-based concept of hypoperfusion-hypodensity mismatch (7). The method is based on evaluating a low net water uptake in native cranial CT. This low net water uptake is considered a marker for the overestimation of the ischemic core in corresponding perfusion imaging (8, 9). We showed that this hypoperfusion-hypodensity mismatch, i.e., the absence of a hypodensity on native CT within the hypoperfused core lesion on perfusion CT, identifies patients within the 4.5-h time window with high accuracy (7, 10). This approach is easily and rapidly applicable with standard radiological software worldwide and without requiring additional software tools (3, 7, 11–13).

We, therefore, performed a further analysis of the MissPerfeCT study and compared the evidence-based method of computed tomography (CT) automated perfusion imaging with the new CT hypoperfusion-hypodensity mismatch approach regarding the accuracy of identifying patients suitable for thrombolysis.

## 2 Methods

### 2.1 Study design and patients

This analysis of the retrospective multicenter MissPerfeCT observational cohort study (08/2009–11/2017) (7) includes consecutive patients with known onset of symptoms from seven tertiary stroke centers who were clinically diagnosed with acute ischemic stroke and received multimodal CT on admission, including standard native cerebral CT (NCCT), CT angiography (CTA), and perfusion CT (CTP). Consecutive patients were included to reduce the risk of bias. Patients from the following university medical centers were included: Bochum (10/2016–08/2017), Goettingen (10/2016–11/2017), Dresden (05/2015–12/2016), Greifswald (09/2015–10/2017), Luebeck (03/2015–12/2016), LMU Munich (08/2009–06/2012), and Muenster (05/2016–11/2017). Inclusion criteria were as follows: 1. evidence of acute intracranial vessel occlusion (any supratentorial proximal or peripheral artery of the ACA, MCA, or PCA territory) by ischemic perfusion deficit and/or CT hyperdense thrombus and/or CTA vessel occlusion, 2. acute symptoms attributable to the ischemic CTP lesion, and 3. sufficient NCCT quality for judgment of early ischemic hypodensity (potential limitations were old infarcts, severe white matter disease, and movement artifacts); sufficient CTP quality for judgment of the ischemic core lesion (potential limitations were insufficient contrast bolus or movement artifacts), and processing by RAPID automated perfusion software.

All datasets of patients fulfilling the inclusion criteria were additionally processed with automated perfusion software (RAPID, RapidAI, Ca, United States), and mismatch criteria were defined according to the Extending the Time for Thrombolysis in Emergency Neurological Deficits (EXTEND) trial criteria (ratio of mismatch >1.2 between CBF < 30% and Tmax >6 s or absolute mismatch >10 mL if total core volume < 70 mL) (3, 11).

The study was approved by the institutional ethics committee (*Ethikkommission der Ärztekammer Westfalen Lippe*; reference number 2017-233-f-S), which waived informed consent because all identifying information was removed before the retrospective analysis. The local ethics committees of all participating centers gave approval according to their local protocol for sharing retrospective and anonymized data. All study protocols and procedures were conducted in accordance with the Declaration of Helsinki. The MissPerfeCT study is a retrospective observational multicenter cohort study and is registered with clinicaltrials.gov (NCT04277728). This study followed the standards for reporting of diagnostic accuracy (STARD) reporting guidelines.

### 2.2 Imaging protocol

Patients received NCCT, CTA, and whole-brain CTP performed on 64 or 128 dual slice scanners (Siemens Definition AS+; Siemens Definition Flash, Siemens Healthcare, Forchheim, Germany; Philips Brilliance 64, Philips Medical Systems, Eindhoven, Netherlands). CT: 120 kV, 280 to 320 mA, 5.0 mm slice reconstruction; CTA: 100 to 120 kV, 260 to 300 mA, 1.0 mm slice reconstruction, 5 mm MIP reconstruction with 1 mm increment; and CTP: 80 kV, 200 to 250 mA, 5 mm slice reconstruction (max. 10 mm), slice sampling rate 1.50 s (min. 1.33 s), scan time 45 s (max. 60 s), biphasic injection with 30 mL (max. 40 mL) of highly iodinated contrast medium with 350 mg iodine/mL (max. 400 mg/mL) injected with at least 4 mL/s (max. 6 mL/s) followed by 30 mL of NaCl chaser bolus. All perfusion parameter maps were calculated on a dedicated workstation (Syngo VE52A with VPCT-Neuro; Siemens Healthcare, Forchheim, Germany) based on a deconvolution model by least mean squares fitting, including cerebral blood volume (CBV), cerebral blood flow (CBF), mean transit time (MTT), and time to drain (TTD) (14, 15).

### 2.3 Image analysis

**Figure 1:**
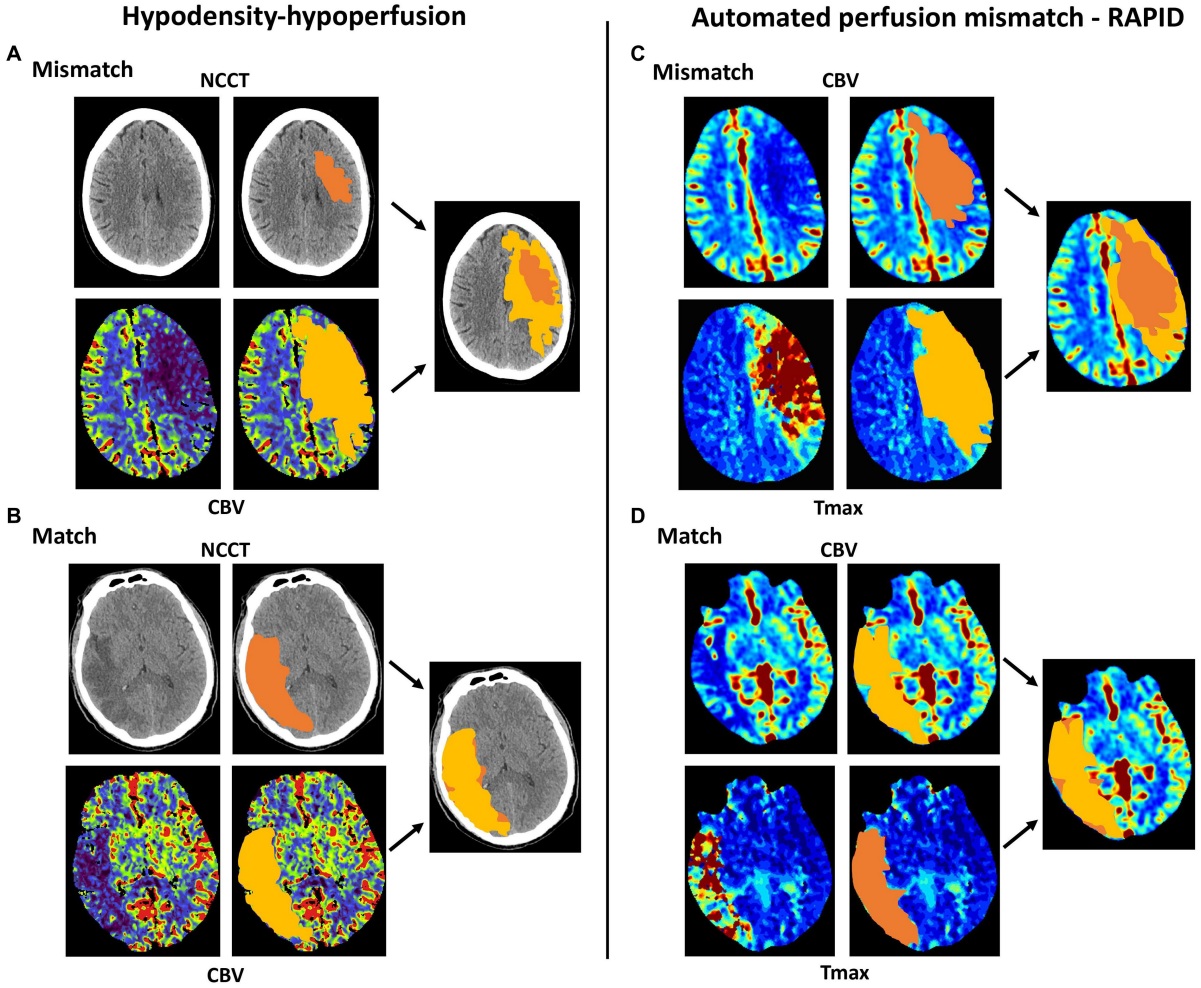
Schematic comparison between CT hypoperfusion-hypodensity mismatch and automated perfusion mismatch (RAPID). **(A)** Presence of hypoperfusion-hypodensity mismatch: An acute ischemic core lesion is readily visible in the cerebral blood volume (CBV) perfusion map, but there is only a small hypodensity on the native cerebral CT (NCCT). **(B)** Absence of hypoperfusion-hypodensity mismatch: A large acute ischemic core lesion is readily visible in the CBV perfusion map, and there is a clear hypodensity on the NCCT. **(C)** Presence of automated perfusion mismatch: An acute ischemic core lesion is readily visible in the Tmax perfusion map, but there is only a small core lesion readily visible in cerebral blood flow. **(D)** Absence of automated perfusion mismatch: A large, overlapping acute ischemic core lesion is visible in the CBV and Tmax perfusion maps.

The algorithm to identify a hypoperfusion-hypodensity mismatch was defined as previously published (Figure 1) (7). It is an easy-to-use method in which two raters, blinded to clinical information, conduct a side-by-side panel comparison. In brief, it consisted of the following steps: First, the total ischemic area was identified by the visual inspection of sensitive MTT or TTD maps. Within this ischemic area of bolus delay, the core lesion, defined as a lesion of high infarct probability, was identified in CBV parameter maps, showing significantly reduced perfusion values of less than 2 mL/ 100 mL or less than 30% relative to the normal side. If no CBV lesion was present, a CBF lesion was used to define the core lesion with significantly reduced perfusion values of less than 30 mL/100 mL/min or less than 60% relative to the normal side. Then, the corresponding region in the NCCT was identified and judged for the presence of a hypodense lesion with respect to the healthy side, consistent with early acute infarct. For this purpose, the NCCT and perfusion CT maps displaying the ischemic core were presented slice by slice because a hypoperfusion-hypodensity match should encompass all slices. The goal was to rate NCCT with high specificity for definite early infarct: in case of doubt as to whether there was a clear hypodensity present, images were rated as an absence of hypodensity. Overall, the judgment about the presence of a CT hypoperfusion-hypodensity mismatch requires 1–2 min.

### 2.4 Statistical analysis

Patients were classified as eligible or not eligible for thrombolysis. Eligibility was given when patients presented within 4.5 h or for patients beyond 4.5 h when a perfusion mismatch was detected with automated perfusion software according to the EXTEND criteria. We compared patients eligible for thrombolysis with those not eligible by using Pearson’s chi-square test for categorical variables and Student’s t-test or the Mann–Whitney U-test for continuous variables. We calculated the area under the curve (AUC), sensitivity, specificity, positive predictive value, and negative predictive value (with Clopper–Pearson 95% confidence intervals) for the identification of patients eligible for iv-tPA. Statistical analyses were carried out using SPSS (version 26).

### 2.5 Data availability statement

The data that support the findings of the study are available from the corresponding author upon request.

## 3 Results

### 3.1 Patient baseline characteristics

A total of 247 patients were included in our study, of whom 219 (88.7%) were suitable for thrombolysis and 28 (11.3%) were not suitable for thrombolysis (comprising patients with an onset <4.5 h and those with an onset >4.5 h fulfilling automated mismatch criteria). The median time from onset to CT was 2 h and 42 min (standard deviation 2 h 18 min) in patients suitable and 8 h 16 min (SD 6 h 6 min) not suitable for thrombolysis. Table 1 shows the patients’ baseline characteristics. Patients suitable for thrombolysis more often had hypertension than those not suitable for thrombolysis. Groups were comparable regarding age, sex, other comorbid conditions, and NIHSS score on admission.

**Table 1:** Baseline characteristics of patients.

	Patients suitable for thrombolysis (*n* = 219)	Patients not suitable for thrombolysis (*n* = 28)	*p*-value
Demographics			
Age, mean (SD), y	73.9 (13.3)	77.3 (15.6)	0.34
Missing, *n*	0	0	
Women, *n* (%)	110 (50.2)	14 (50.0)	0.98
Missing, *n*	0	0	
Comorbidities, *n* (%)			
Hypertension	154 (77.8)	12 (46.2)	< 0.001
Missing, *n*	21	2	
Diabetes mellitus	38 (19.2)	5 (19.2)	1.00
Missing, *n*	21	2	
Atrial fibrillation	87 (43.9)	13 (50.0)	0.56
Missing, *n*	21	2	
Hypercholesterolemia	77 (43.3)	8 (33.3)	0.36
Missing, *n*	41	4	
Coronary heart disease	50 (34.7)	3 (13.6)	0.05
Missing, *n*	75	6	
Smoker	44 (22.1)	4 (16.7)	0.45
Missing, *n*	24	3	
NIHSS score			
Median (IQR)	7.0 (8.0)	8.5 (9.8)	0.32
Mean (SD)	8.6 (5.9)	10.0 (6.8)	0.26
Missing, *n*	8	0	
Time from symptom onset to CT, mean, (SD), hr.:min	2:42 (2:18)	8:16 (6:06)	< 0.001
Missing, *n*	0	0	

Legend: SD, standard deviation; IQR, interquartile range; NIHSS, National Institutes of Health Stroke Scale. 3.2% missing for NIHSS.

### 3.2 Presence of hypoperfusion-hypodensity mismatch or automated perfusion mismatch

Of 219 patients suitable for thrombolysis, 197 (90.0%) were presented within 4.5 h and 22 (10.0%) beyond 4.5 h (Table 2). Of the 197 patients within 4.5 h of symptom onset, 190 (96.4%) were identified by the presence of hypoperfusion-hypodensity mismatch and 88 (44.7%) by automated perfusion mismatch. Of the 22 patients presenting beyond 4.5 h classified as suitable for thrombolysis by automated perfusion mismatch, 5 (22.7%) were also identified by hypoperfusion-hypodensity mismatch. There were 28 patients who presented beyond 4.5 h and were classified as not suitable for thrombolysis by automated perfusion analysis. Within this group, eight patients (28.6%) were likewise classified as not suitable for thrombolysis by hypoperfusion-hypodensity mismatch.

**Table 2:** Presence of hypoperfusion-hypodensity mismatch or automated perfusion mismatch.

	Patients suitable for thrombolysis			Patients not suitable for thrombolysis		
Imaging modality	< 4.5 h *n* = 197	> 4.5 h *n* = 22	*p*	< 4.5 h *n* = 0	> 4.5 h *n* = 28	*p*
Patients identified by hypoperfusion-hypodensity mismatch, *n* (%)	190 (96.4)	5 (22.7)	< 0.001	0 (0.0)	8 (28.6)	N/A
Patients identified by automated perfusion mismatch, *n* (%)	88 (44.7)	22 (100)	< 0.001	0 (0.0)	0 (0.0)	N/A

Using hypoperfusion-hypodensity mismatch to classify patients suitable for thrombolysis yielded a sensitivity of 89.0% (95% CI 84.1–92.9), a specificity of 71.4% (95%CI 51.3–86.8), a positive predictive value of 96.1% (95%CI 92.3–98.3), and a negative predictive value of 45.5% (95%CI 30.4–61.2) (Table 3). Automated perfusion mismatch quantification identified patients suitable for thrombolysis with 50.2% (95%CI 43.4–57.9) sensitivity, 100.0% (95%CI 57.7–100.0) specificity, 100.0% (95%CI 96.7–100.0) positive predictive value, and 20.4% (95%CI 14.0–28.2) negative predictive value.

**Table 3:** Sensitivity, specificity, and predictive values for the identification of patients suitable for thrombolysis.

Predicting factors	Sensitivity, % (95% CI)	Specificity, % (95% CI)	Positive predictive value, % (95% CI)	Negative predictive value, % (95% CI)	AUC (95% CI)
Hypoperfusion-hypodensity mismatch	89.0 (84.1–92.9)	71.4 (51.3–86.8)	96.1 (92.4–98.3)	45.5 (30.4–61.2)	80.2 (70.1–90.4)
Automated perfusion mismatch	50.2 (43.4–57.0)	100.0 (57.7–100.0)	100.0 (96.7–100.0)	20.4 (14.0–28.2)	75.1 (68.3–82.0)

AUC, area under the curve; CI, confidence interval.

## 4 Discussion

This multicenter study shows that the assessment of hypoperfusion-hypodensity mismatch identifies patients who are eligible for thrombolysis with higher sensitivity (89.0%) compared with automated perfusion analysis (50.2%) and might thus increase the number of patients treated with thrombolysis among those with unknown time of symptom onset. Of note, the positive predictive value was also very high with the assessment of hypoperfusion-hypodensity mismatch (96.1%), even though it was naturally lower than with automated analysis, where patients with a favorable imaging profile were defined as “ground truth” for eligibility for thrombolysis in this study, inevitably leading to specificity and positive predictive value of 100%. For hypoperfusion-hypodensity mismatch evaluation, the false negative rate was 54.4%, and for automated mismatch analysis, it was 79.6%. This implies that patients eligible for thrombolysis may occasionally be missed by both methods, but less frequently with hypoperfusion-hypodensity mismatch. Thus, in a real-world setting, our approach is likely to perform even better than in this study. The concept of hypoperfusion-hypodensity mismatch is based on the pathophysiology of cerebral ischemia, i.e., the uptake of water into the ischemic brain (10). This tissue water uptake after cerebral artery occlusion follows a characteristic course that is visualized by a decreasing CT density within the hypoperfused brain region (16, 17). We have recently shown that the hypoperfused region and the region of hypodensity usually match after 4.5 h and vice versa, patients without such a match, i.e., a hypoperfusion-hypodensity mismatch, are suitable for thrombolysis (7). However, from our previous study, it was not clear how this approach performs compared to the automated assessment of perfusion mismatch that was validated in the randomized EXTEND trial (3, 13). The present study shows that hypoperfusion-hypodensity mismatch increases the proportion of patients suitable for thrombolysis among those with an unknown time of symptom onset compared to automated perfusion mismatch quantification. This finding is in line with a recent MRI study that found that the yield of a tissue clock imaging approach to select patients eligible for thrombolysis in an unknown time window was double that of ischemic core–perfusion mismatch-based patient selection (13). One might argue that a tissue clock approach, such as the hypoperfusion-hypodensity mismatch method, might miss patients beyond 4.5 h who are suitable for thrombolysis. However, previous studies suggest that the majority of patients with unknown time of stroke onset have had their symptom onset within 4.5 h (16, 17). Thus, the number of patients identified by hypoperfusion-hypodensity mismatch presumably outnumbers patients identified by automated perfusion mismatch quantification. In addition, the present study showed that of the 22 patients with symptom onset beyond 4.5 h who were suitable for thrombolysis, as identified by automated perfusion mismatch, 5 patients were also identified by hypoperfusion-hypodensity mismatch. The low proportion of patients with symptom onset beyond 4.5 h in our study might have contributed to the high positive predictive value. The imbalance between patients with symptom onset within 4.5 h and beyond should therefore be acknowledged as a limitation. Nevertheless, the high proportion of patients with symptom onset within 4.5 h reflects the real-world situation, as previous studies showed that a majority of wake-up stroke patients are very likely to be in the 4.5 h time window (18). A further potential weakness of our method is that it detects only a small proportion of patients with symptom onset of more than 4.5 h who are suitable for thrombolysis. However, the majority of patients with wake-up stroke (i.e., patients with unknown time of symptom onset) are very likely to be within the 4.5-h time window (18). It is worth noting that patients with hypertension and coronary artery disease are more likely to be eligible for thrombolysis. Although observed in previous studies, the reason remains unclear (7). One possible explanation could be that patients with a history of cardiovascular disease may be more vigilant regarding acute cardiovascular events.

The multicenter, randomized, double-blind WAKE-UP trial confirmed the rationale of the “tissue clock” approach for the identification of stroke patients with unknown time of symptom onset eligible for thrombolysis (6). Following this concept, patients with visible changes on DWI but normal FLAIR are likely within the time window of thrombolysis. The WAKE-UP trial showed that thrombolysis in patients with unknown time of symptom onset guided by MRI DWI-FLAIR mismatch results in a significantly better functional outcome (6). However, compared with MRI, CT is less affected by contraindications (such as pacemakers) and is more generally available in the acute setting in most hospitals that treat acute ischemic stroke patients, and thus is the primary imaging modality used globally.

Overall, the hypoperfusion-hypodensity mismatch method has several advantages over existing imaging-based methods for the identification of patients with unknown time of symptom onset who are eligible for thrombolysis, including the speedy accessibility of computed tomography worldwide compared to MRI and the dispensability of automated software tools. A limitation of our study is its retrospective design. However, all images were assessed by readers blinded to clinical information.

## 5 Conclusion

Eligibility for thrombolysis among stroke patients with unknown time of symptom onset can be determined by the detection of a hypoperfusion-hypodensity mismatch with higher sensitivity compared to automated perfusion mismatch quantification, the gold standard that was established in a randomized trial.

Thus, the simple method of hypoperfusion-hypodensity mismatch can potentially increase the proportion of patients with unknown time of stroke onset who are treated with thrombolysis.

## Data availability statement

The raw data supporting the conclusions of this article will be made available by the authors, without undue reservation.

## Ethics statement

The studies involving humans were approved by Ethikkommission der Ärztekammer Westfalen Lippe (reference number: 2017-233-f-S). The studies were conducted in accordance with the local legislation and institutional requirements. Written informed consent for participation was not required from the participants or the participants' legal guardians/next of kin in accordance with the national legislation and institutional requirements.

## Author contributions

PSp: Formal analysis, Visualization, Writing – review & editing, Conceptualization, Investigation, Writing – original draft, Methodology, Project administration. AK: Formal analysis, Visualization, Conceptualization, Investigation, Methodology, Writing – review & editing. LM: Visualization, Writing – review & editing, Formal analysis, Investigation, Methodology, Data curation. CK: Formal analysis, Investigation, Methodology, Writing – review & editing. VP: Formal analysis, Investigation, Methodology, Writing – review & editing. KT: Formal analysis, Investigation, Methodology, Writing – review & editing. MD: Formal analysis, Investigation, Methodology, Writing – review & editing. CL: Formal analysis, Investigation, Methodology, Writing – review & editing. DK: Formal analysis, Investigation, Methodology, Writing – review & editing. SL: Formal analysis, Investigation, Methodology, Writing – review & editing. AB: Investigation, Methodology, Writing – review & editing, Formal analysis. LR: Formal analysis, Investigation, Methodology, Writing – review & editing. WK: Formal analysis, Investigation, Methodology, Writing – review & editing. CB: Formal analysis, Investigation, Methodology, Writing – review & editing. WH: Formal analysis, Investigation, Methodology, Writing – review & editing. JF: Formal analysis, Investigation, Methodology, Writing – review & editing. PSc: Formal analysis, Investigation, Methodology, Writing – review & editing. HW: Formal analysis, Investigation, Methodology, Writing – review & editing. HM: Formal analysis, Investigation, Methodology, Writing – review & editing. MP: Investigation, Methodology, Writing – review & editing, Formal analysis. JM: Conceptualization, Investigation, Methodology, Writing – review & editing, Formal analysis.
